# Ferrous Illusion: A Unique Case of Welding Fume Particles Appearing as Metallic Artifacts in MRI

**DOI:** 10.7759/cureus.47404

**Published:** 2023-10-20

**Authors:** Mariam Malik, Rana Bilal Idrees, Jawairia Arif

**Affiliations:** 1 Radiology, Atomic Energy Cancer Hospital, Nuclear Medicine, Oncology and Radiotherapy Institute (NORI), Islamabad, PAK; 2 Radiology, INMOL (Institute of Nuclear Medicine & Oncology) Cancer Hospital, Lahore, PAK

**Keywords:** general radiology, brain anatomy, mri images, metallic artifact, neuro radiology

## Abstract

A rare cause of metallic artifacts over the scalp on magnetic resonance imaging (MRI) is welding fume particles that contain paramagnetic iron oxide particles. These introduce distortion of the magnetic field homogeneity and result in susceptibility artifacts. They may erroneously be reported as a pathology such as calcified lesions; therefore, awareness among radiologists is required. We report a case of a 52-year-old male, an industrial inspector by profession, who presented to the neurology clinic with headaches for which an MRI of the brain without contrast was advised. There was no brain parenchymal signal abnormality; however, numerous small rounded altered signal foci were identified along the scalp, especially in the vertex region, which returned central hypointense and marginal hyperintense signal on all sequences. The imaging signals were suspicious for calcified scalp lesions, and the patient was recalled for clinical examination, which was unremarkable for cutaneous or subcutaneous abnormality on the scalp or elsewhere over the body. A detailed history was taken retrospectively, revealing that the patient had walked through a room where welding was being done before presenting for an MRI exam, without taking a shower. The various altered signal foci over the scalp on MRI based on their shape were hence identified as welding fume particles. These were fine enough not to be visible by the naked eye but determined by the MRI machine because of their magnetic susceptibility artifact. We aim to increase radiologists' awareness of such artifacts that may be seen in patients with occupational exposure to these particles to avoid misdiagnosis of other pathologies.

## Introduction

Magnetic resonance imaging (MRI) is a non-invasive cross-sectional imaging technique with potential benefit of no ionizing radiation exposure [1]. However, it is essential that the technologist performing the study, as well as the reporting radiologist, is well aware of relative and absolute contraindications of the study, especially for patient safety. Assessment of compatibility of medical devices is also necessary to avoid untoward side effects of the procedure [2]. Even though MRI has a high soft tissue contrast, degradation of the image quality may be caused by artifacts.

An imaging artifact refers to false anatomy or pathology that is otherwise not present in the body. The artifacts in MRI can be technique-related or caused by foreign bodies near the patient's body or within the body at the time of scanning. These may deteriorate the image quality or introduce false pathology in images [3]. Metallic objects can result in signal dropout, failure of fat suppression, and geometric distortion [4], or be seen as an area of hypointense signal bordered by a hyperintense rim [1] due to disturbances in the magnetic field homogeneity secondary to their inherent high magnetic susceptibility. These are significantly more pronounced in gradient recalled echo sequence (GRE), which lacks the 180-degree pulse of the spin echo sequences.

Metallic artifacts in brain imaging may be introduced by several objects, including metal drill materials used in craniotomy, surgical wires and sutures of previous surgery, dental grafts, and cosmetics such as eyeliner or hair dye. A less commonly identified cause of artifacts over the scalp is welding fume particles that may be seen in welders and other professionals with potential exposure to these fume particles, the principal constituent of which is iron oxide [5], which has paramagnetic properties. We report this rare case of metallic artifacts over a patient's scalp who passed by a room where welding was being done before coming for an MRI examination.

## Case presentation

**Figure 1:**
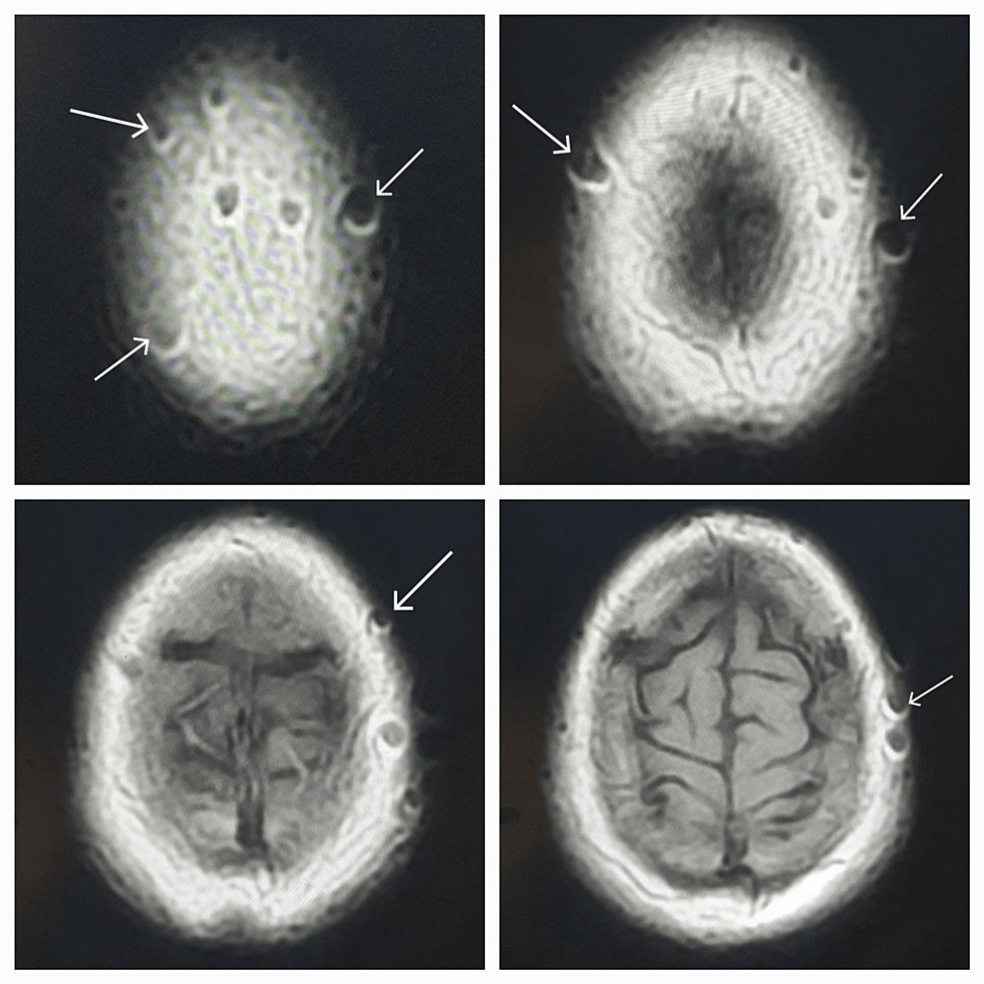
Axial T1 images show multiple bubble-shaped, central low signal, marginal high signal, altered signal foci over the scalp.

**Figure 2:**
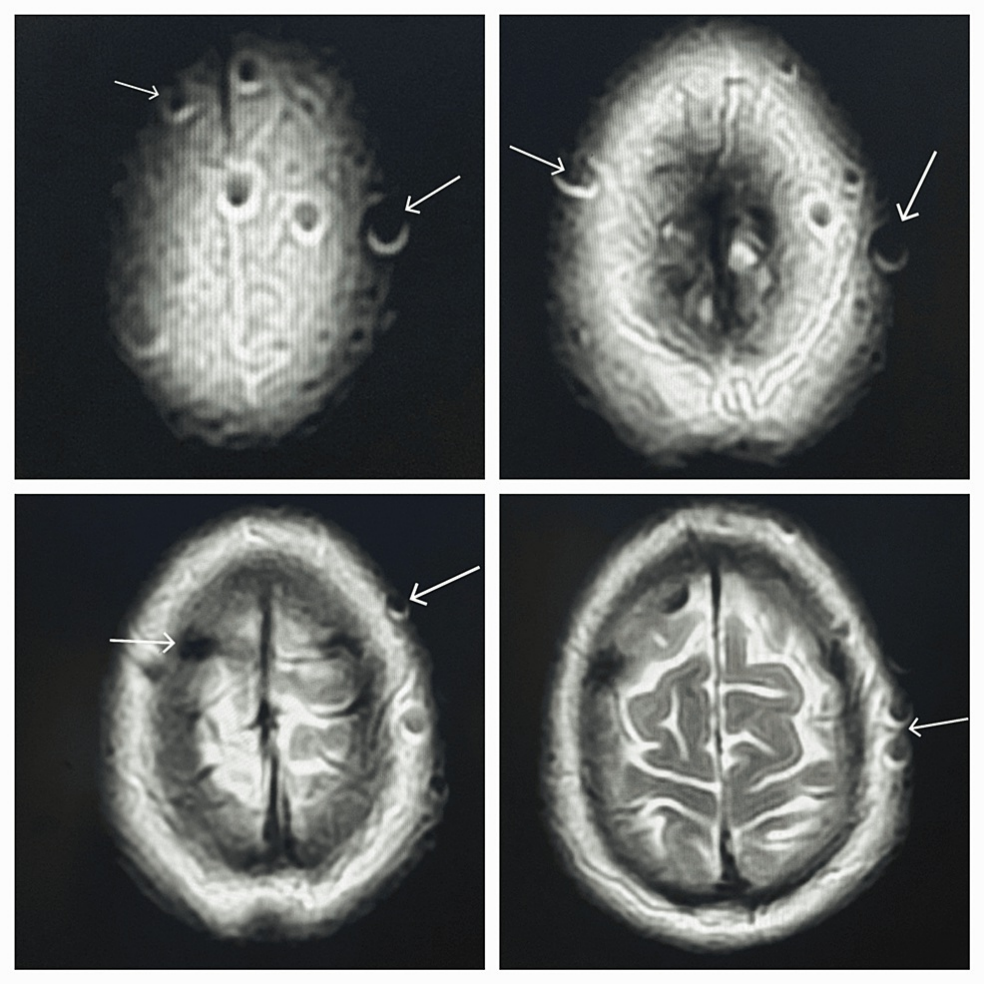
Axial T2 sections at the same levels show the artifacts over the scalp with a central hypointense and marginal hyperintense signal.

**Figure 3:**
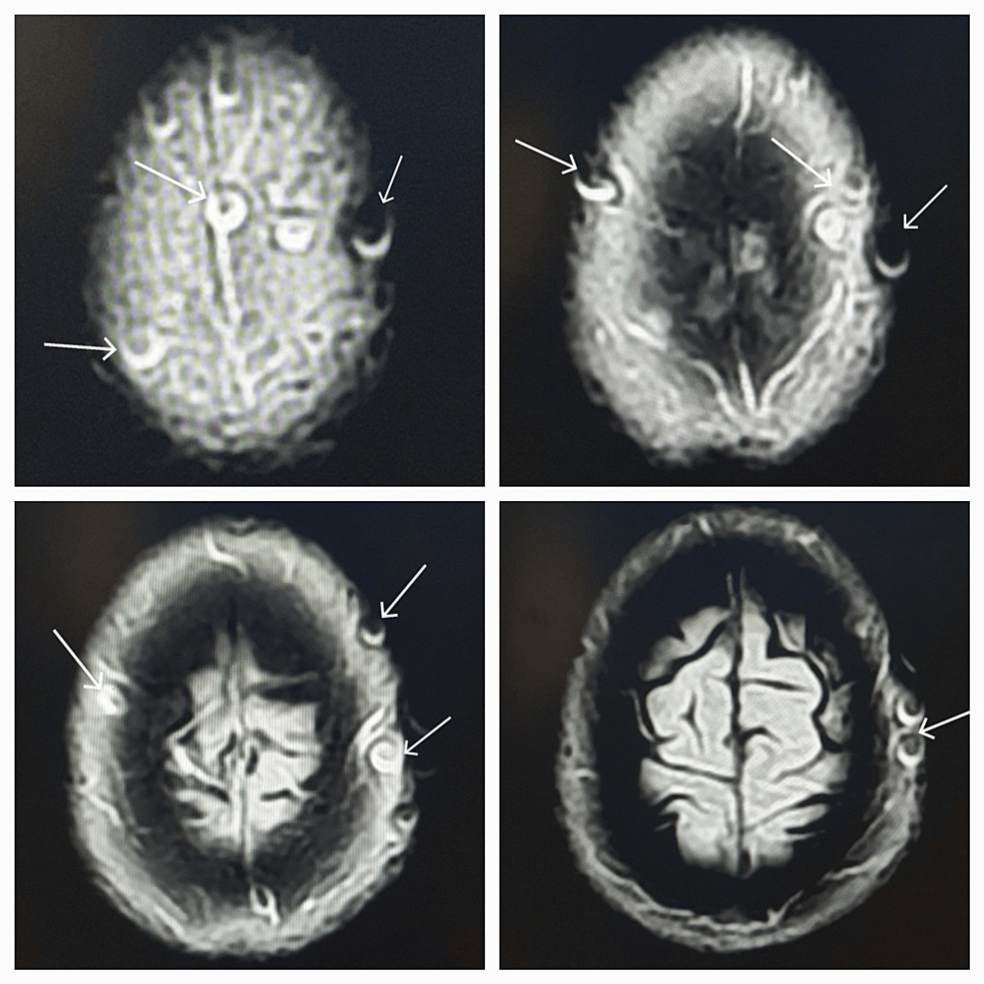
The artifacts are seen with central low and peripheral high signals within the scalp on the axial FLAIR sequence FLAIR: fluid attenuation inversion recovery

We report a rare case of a 52-year-old male, an industrial inspector by profession, who presented to the neurology clinic for headaches. His general physical and neurological examination was unremarkable; however, since the episodes of headaches were frequent, the neurologist advised an MRI brain without contrast to exclude any intracranial pathology. The patient came to the radiology department for an MRI brain performed on a 70cm wide bore Optima™ 450W 1.5 Tesla MRI machine (General Electric Company, Boston, Massachusetts, United States). T1, T2, fluid attenuation inversion recovery (FLAIR), diffusion-weighted imaging (DWI), and apparent diffusion coefficient (ADC) images were acquired as per the departmental protocols. There was no acute infarct, intracranial hemorrhage, mass effect, or midline shift. No intracranial abnormal signal mass or brain parenchymal signal abnormality was identified. However, numerous rounded altered signal foci of sizes measuring up to 1.5 cm were identified along the scalp, especially in the region of the vertex, which returned central hypointense and marginal hyperintense signals on all sequences (Figures 1-3 ).

**Figure 4:**
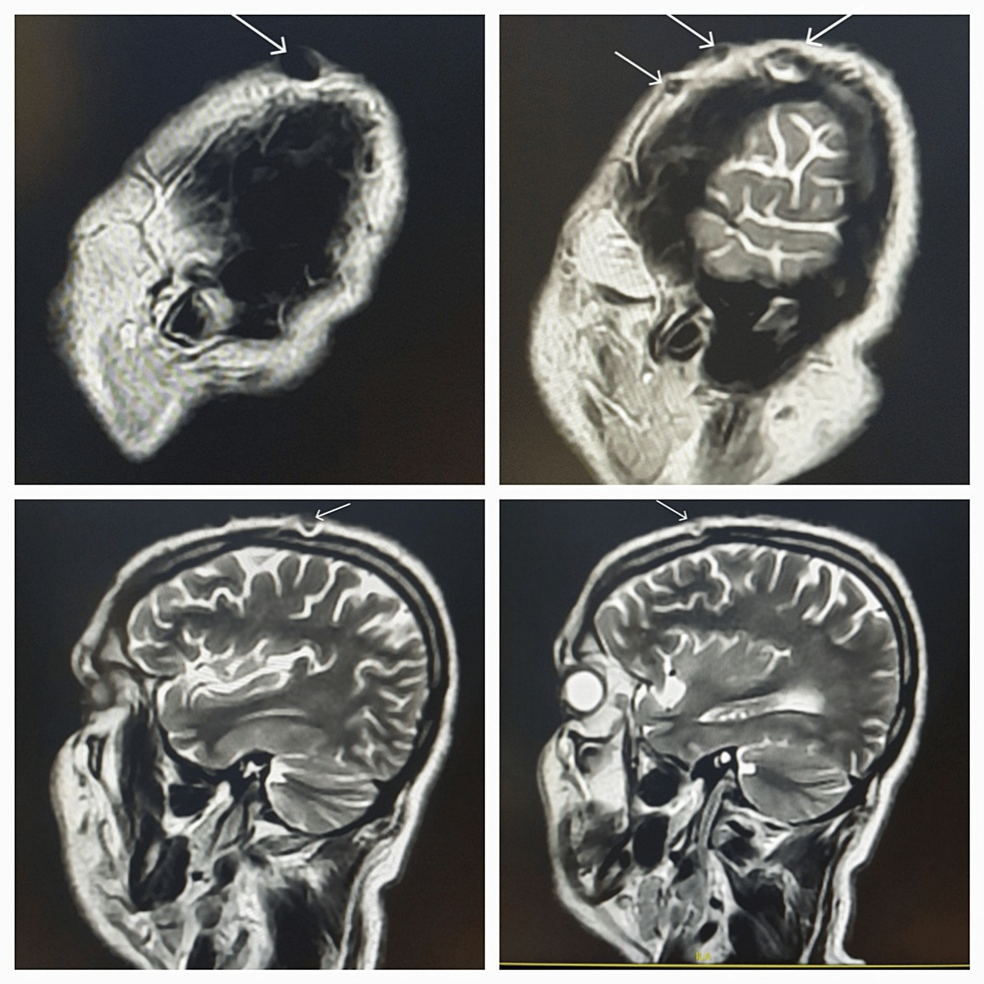
Sagittal T2 images demonstrate the artifacts over the scalp, which at places appear to be gently scalloping the cortical outlines of the skull.

The imaging signals were suspicious for calcified scalp lesions as the signal remained low on multi-sequential imaging. Hence, the patient was recalled for a clinical examination of the scalp. Detailed examination revealed no cutaneous or subcutaneous abnormality on the scalp or elsewhere over the body. A detailed history was taken retrospectively, revealing that the patient had walked through a room where welding was done before the MRI exam. The patient also did not take a shower before presenting to us. The various altered signal foci over the scalp on MRI based on their shape were hence identified as welding fume particles. These were fine enough not to be visible by the naked eye but identified by the MRI machine because of their magnetic susceptibility artifact (Figure 4).

## Discussion

The classification of an artifact into mild, moderate, or severe depends on its size and the matter responsible for its introduction in the image. A size smaller than the size of the material classifies it as mild, the same size as the material categorizes it as moderate, and more significant than the object is identified as severe [6]. Moreover, the size of the artifact is determined by the category and thickness of magnetic material, its contours and orientation, and the static magnetic field strength, as the bandwidth of the receiver coils [1]. There is a division of metallic objects according to their magnetic susceptibility into ferromagnetic, paramagnetic, and diamagnetic, where ferromagnetic materials are intensely magnetized in magnetic fields, and paramagnetic matter is moderately magnetized. In contrast, diamagnetic bodies show faint magnetization [7]. Hence, ferromagnetic substances possess the highest potential of causing artifacts, while diamagnetic implications are less likely to cause substantial artifacts in imaging [8].

**Figure 5:**
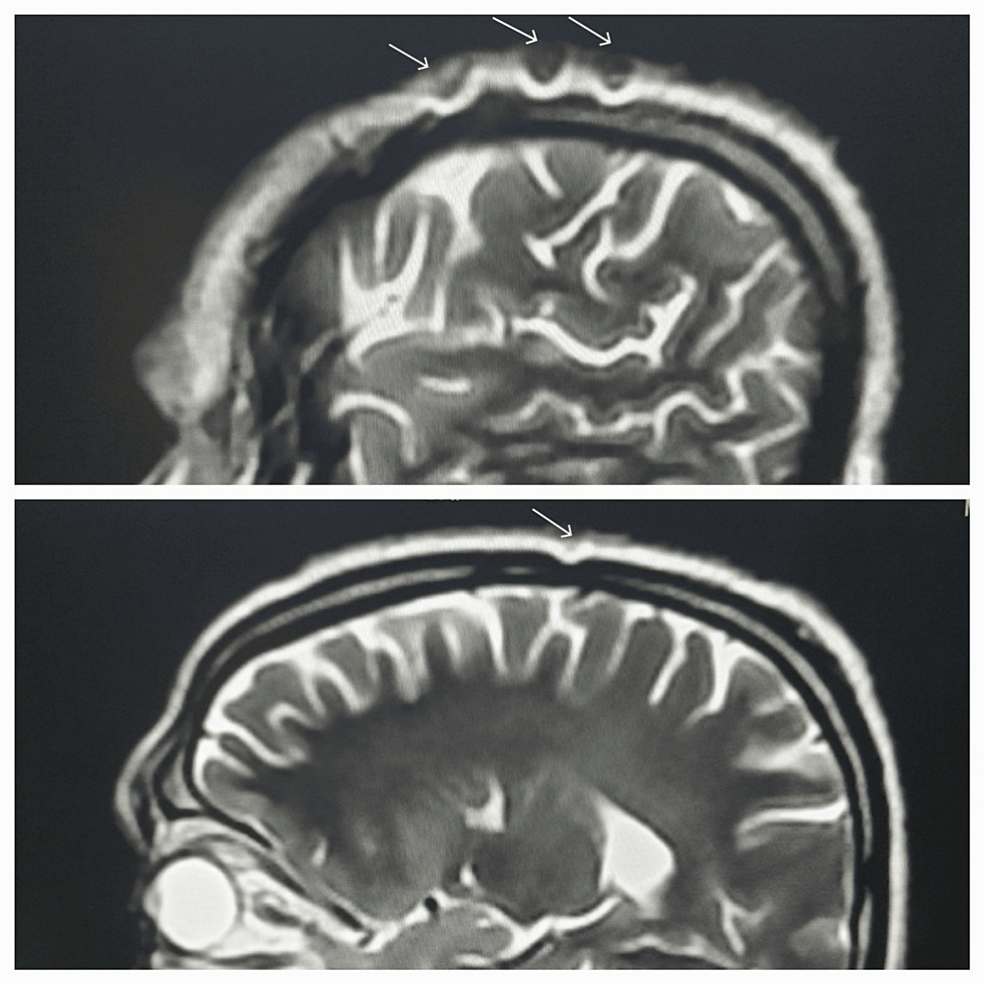
Sagittal T2 images showing the artifacts within the scalp, which mimic post-surgical defects at places.

The cause of metal artifacts in the imaging of our patient is attributable to paramagnetic iron oxide contents, which influence the magnetic field homogeneity. The fume particles become magnetically susceptible and result in associated geometric distortion. These may go unnoticed by the patients and are too small to be visible on other imaging modalities such as radiography or computed tomography (CT). However, they are discretely seen on MRI. Hence, multiple artifacts observed within the scalp, especially along the vertex in our patient, are susceptibility artifacts with a size larger than the welding fume particles and an appearance similar to the post-surgical defect (Figure 5), a finding consistent with the study of Somasundaram et al. [9].

It is vital that these be correctly recognized and not reported as pathology in the scalp, in which case detailed history and clinical examination are crucial to exclude other possibilities.

## Conclusions

Awareness amongst radiologists of the susceptibility artifacts that may be caused by paramagnetic iron oxide particles of welding fumes is essential to avoid misdiagnosing other pathologies in patients with occupational exposure to such particles. This is especially important as calcified lesions can appear as similar signals on MRI and therefore these artifacts may be misinterpreted as a pathology. Radiologists must take a detailed history and clinically examine the patients in cases of doubt to exclude possible differential mimickers. Our case hi lights this unique example that must be kept in mind while reporting MRI images. 
